# Iron Overload Induces Oxidative Stress, Cell Cycle Arrest and Apoptosis in Chondrocytes

**DOI:** 10.3389/fcell.2022.821014

**Published:** 2022-02-18

**Authors:** Asima Karim, Khuloud Bajbouj, Jasmin Shafarin, Rizwan Qaisar, Andrew C. Hall, Mawieh Hamad

**Affiliations:** ^1^ Department of Basic Medical Sciences, College of Medicine, University of Sharjah, Sharjah, United Arab Emirates; ^2^ Sharjah Institute for Medical Research, University of Sharjah, Sharjah, United Arab Emirates; ^3^ Edinburgh Medical School, Biomedical Sciences, College of Medicine and Veterinary Medicine, The University of Edinburgh, Edinburgh, United Kingdom; ^4^ Department of Medical Laboratory Sciences, College of Health Sciences, University of Sharjah, Sharjah, United Arab Emirates

**Keywords:** iron overload, osteoarthritis, C-20/A4 cells, apoptosis, oxidative stress, chondrocytes

## Abstract

Clinical and experimental evidence point to the presence of considerable links between arthropathy, osteoarthritis (OA) in particular, and iron overload possibly due to oxidative stress and tissue damage. However, the specific cellular targets of iron overload-related oxidative stress in OA remain ambiguous**.** We examined the effects of iron overload on chondrocyte health using the C-20/A4 chondrocyte cell line. Cells were treated with increasing concentrations of ferric ammonium citrate (FAC) to mimic iron overload *in vitro*. Treated cells were assessed for cell viability, cycling, apoptosis, collagen II synthesis, and oxidative stress along with cellular iron content and the expression of key iron regulatory genes. FAC treatment resulted in an increase in ferritin expression and a significant decrease in the expression of hepcidin, ferroportin, transferrin receptors 1 (TfR1) and TfR2. Increased labile iron content was also evident, especially in cells treated with high FAC at 24 h. High doses of FAC treatment also induced higher levels of reactive oxygen species, reduced collagen II production, disrupted cell cycle and higher cell death as compared with untreated controls. In conclusion, findings presented here demonstrate that iron overload disrupts cellular iron homeostasis, which compromises the functional integrity of chondrocytes and leads to oxidative stress and apoptosis.

## Introduction

Osteoarthritis (OA) is a common joint disease which typically causes severe pain and disability (Vina and Kwoh, 2018). OA is characterized by cartilage degeneration and subchondral bone remodeling (Loeser et al., 2012). Old age (Li et al., 2013; Loeser, 2017) and disrupted iron homeostasis (Burton et al., 2020) are believed to be significant contributing factors in the initiation and/or exacerbation of OA (Nugzar et al., 2018). Hereditary hemochromatosis (HH), which associates with excessive iron accumulation in body tissues and organs due to the inheritance of one or more mutations in the Human homeostatic iron regulator (HFE) gene, has been shown to lead to OA-like arthropathy (Carroll et al., 2011). Nearly two-thirds of HH patients tend to develop OA-like symptoms (Inês et al., 2001), often with radiological features that resemble idiopathic primary OA (Dallos et al., 2010). The presence of high ferritin levels and excessive iron accumulation has been documented in the synovial fluid and articular cartilage/synovial membranes of HH patients showing signs of OA (Dejaco et al., 2017; Simão et al., 2019). In articular cartilage, enhanced production of matrix degrading enzymes such as matrix metalloproteinases (MMPs) and the “a disintegrin-like and metalloprotease domain with thrombospondin type 1 repeats” (ADAMTS) (Camacho et al., 2016) and reduced matrix production/aggrecan levels have also been associated with high iron levels (Simão et al., 2019).

Abnormal accumulation of iron in the immediate vicinity of joint structures as observed in HH and old age may directly or indirectly lead to osteochondral damage (Kennish et al., 2014). A strong correlation between iron overload and cartilage degeneration leading to OA has been documented (Burton et al., 2020; Ferreira et al., 2021). Besides HH, degenerative cartilage changes and OA-like arthropathy have been reported in other clinical conditions such as haemophilia and thalassemia (Roosendaal et al., 1999; Economides et al., 2012) where joint structures are usually exposed to high systemic iron levels. However, chelation of excessive systemic iron does not improve the symptoms of joint arthropathy (Husar-Memmer et al., 2014), suggesting that joint damage under conditions of iron overload relates more to iron sequestered in the joint tissues rather than systemic iron levels. In support of this possibility, knee joint synovial membranes in hemochromatosis patients often show significant levels of iron deposition although systemic iron levels are normal (Chehade and Adams, 2019). Given its large molecular mass (>470 kDa), the iron storage protein ferritin is unlikely to pass from circulation to the synovial cavity (Chiou and Connor, 2018). Therefore, it is possible that high ferritin levels in the synovial fluid are reflective of *in situ* ferritin overproduction rather than systemic iron influx (Chehade and Adams, 2019). Elevated synovial ferritin levels are also associated with the OA phenotype in aging and iron overload-related conditions (Carroll et al., 2010; Kennish et al., 2014).

The general understanding of how excess iron initiates and/or exacerbates joint damage notwithstanding, detailed knowledge of how excess iron affects the metabolic activity and viability of chondrocytes is still lacking. Additionally, whether chondrocytes can respond to changes in cellular iron content in such a way that protects against cell damage and death has yet to be investigated. In this study, we investigated the effects of cellular iron overload on chondrocyte viability, cellular iron content and the expression status of key iron regulatory genes using the ferric ammonium citrate (FAC)-treated human chondrocyte cell line C-20/A4 as a model.

## Materials and Methods

### Cell Cultures and Treatment Protocol

The immortalized human juvenile costal chondrocyte C-20/A4 cell lines (a kind gift from Dr. Mary B. Goldring, Hospital for Special Surgery, NYC, United States and Dr. Janine Post, Developmental BioEngineering/TechMed Center/University of Twente, Netherlands) were used in this study. Under aseptic conditions, cells were cultured in Dulbecco’s Modified Eagle’s Medium (DMEM) high glucose (ThermoFisher; Inc., Waltham, Massachusetts, United States) supplemented with 10% fetal calf serum (FCS) (Sigma, St. Louis, MO, United States) and penicillin/streptomycin (1000 U/10 mg/ml; Sigma) at 37°C with 5% CO_2_. The cells were seeded at 10,000-20,000 cells/cm^2^ in 25 cm flasks. While reaching ∼70% confluency, cells were treated with ferric ammonium citrate (FAC) [TC453 FAC; Brown cell culture tested (Sigma-Aldrich)], at various concentrations of 2–300 µM dissolved in phosphate-buffered saline (PBS; Sigma). The FAC solution was prepared fresh and filtered through 0.22 µm syringe filter under sterile conditions. The cell cultures were maintained for 24 and 48 h with FAC treatment prior to collection and analysis. The control flasks were left untreated with standard culture medium.

### 3-(4,5-Dimethylthiazol-2-yl)-2,5- Diphenyltetrazolium Bromide (MTT) Cell Viability Assay

An MTT colorimetric assay (Sigma-Aldrich) was used to assess C-20/A4 cell viability in control and FAC treated groups. In 96-well plates, chondrocytes were seeded at a density of ∼93,000 cells/cm^2^. The cells were provided an appropriate seeding time of ∼24 h for adherence. The cells were then treated with increasing concentrations of FAC for 24 and 48 h. At specific time points post culture, MTT salt was mixed with cells and incubated at 37°C for 2 h in a humidified CO_2_ incubator at 5% CO_2_. MTT formazan product was dissolved in dimethyl sulfoxide (DMSO) and absorbance was read at 570 nm on a microplate reader. The assay was repeated for three independent experiments and the results analysed.

### Reactive Oxygen Species Assay

The ROS levels in C-20/A4 chondrocytes were measured by flow cytometry using the Total ROS Assay Kit (cat # 88-5930–74; ThermoFisher; Inc., Waltham, Massachusetts, United States). Cells were either untreated (control group) or treated with 200 or 300 µM FAC for 24 and 48 h. At each time point, total ROS levels were measured in live unfixed cells by using the kit as per the manufacturer’s instructions and analysed by flow cytometry (Becton Dickinson, United States). Flow cytometry data was analysed using FlowJo software with the Watson pragmatic model (Tree Star, Ashland, OR, United States).

### Western Blotting Analysis

Untreated and FAC treated chondrocytes were collected after 24 and 48 h of culture and lysed in ice-cold NP-40 lysis buffer (1.0% NP-40, 150 mM of NaCl, 50 mM of Tris-Cl, pH 8.0) containing protease cocktail inhibitor tablets (Cat. No. S8830; Sigma, Germany). Using the standard Bradford method (Cat. No. 500-0006; Bio-Rad, Hercules, CA, United States) the lysate was quantified for protein concentrations and measurements performed by spectrophotometer. Lysate aliquots containing 50 µg of protein were separated by 15% sodium dodecyl sulfate–polyacrylamide gel electrophoresis (SDS-PAGE) and transferred onto a nitrocellulose membrane (Cat. No. 1620112; Bio-Rad). The membrane was then blocked by 5% skimmed milk powder for 1 h at room temperature, washed with (TBST), and reacted with primary immunoglobulin G (IgG) unlabelled antibodies at 1:1,000 dilution overnight at 4°C. The primary antibodies used were anti-FTN: (Cat No. aa154-183) from LifeSpan Biosciences, Seattle, Washington; anti-HEP: (Cat. No. ab57611); anti-FPN: (Cat No ab85370); anti-TfR1: (Cat No. ab84036); anti-TfR2: (Cat No. ab84287); anti-Collagen II: (Cat No. ab185430); all from Abcam, Cambridge, United Kingdom. Secondary anti-mouse (Cat. No. 7076; Cell Signalling Technology, Anvers, MA, United States) was reacted with the membrane at 1:1,000 dilutions for 1 h at room temperature, and the secondary anti-rabbit antibody (Cat. No. 97040; Abcam) was reacted with the membrane at 1:5000 dilution for 1 h at room temperature. Chemiluminescence was detected using the ECL kit (Cat. No. 32106; Thermo-Scientific). Protein band quantification was carried out using the Bio-Rad Image Lab software (ChemiDoc™ Touch Gel and Western Blot Imaging System; Bio-Rad, Hercules, CA, United States). β-actin was used as a loading and normalization control, and the values of control samples were defined as 1.0, the values of treated samples were quantified relative to that of control and normalized against β-actin.

### Assessment of Cellular Labile Iron by Flow Cytometry

Labile intracellular iron content was measured in control (untreated) and treatment groups of chondrocytes by calcein staining method modified and described by authors elsewhere (Bajbouj et al., 2017). Briefly, cells were washed twice with PBS; overall, 0.5 × 10^6^ cells were incubated for 15 min at 37°C in the presence of 0.5 µM of calcein acetoxymethyl ester (CA-AM; Cat. No. 56496, Sigma-Aldrich). Cells were then washed twice and treated with DFO (Novartis, Switzerland) at 100 µM. Cells were analysed by flow cytometry (Becton Dickinson, United States) at a rate of 1,000 events/s applying a 488-nm laser beam for excitation. Untreated cells cultured under the same conditions served as negative controls. A minimum of 30,000 events were collected/sample and percentage positive staining was computed to the 99% level of confidence. Mean fluorescence intensity (MFI) as presented here represents the geometric MFI of a log-normal distribution of fluorescence signals. Given that MFI increases as free iron content decreases, a qualitative measure of the change in labile iron pool (LIP) is calculated as ΔMFI (MFI CA-AM/DFO—MFICA-AM alone) where ΔMFI >0 indicates LIP availability, while ΔMFI ≤0 indicates LIP depletion. All flow cytometric data were analysed using the FlowJo software with the Watson pragmatic model (Tree Star, Ashland, OR, United States).

### Cell Cycle Progression Analysis

Cell cycle analysis was performed as described previously (Bajbouj et al., 2017). Briefly, cells were seeded at a density of 210,000 cells/cm^2^. Cells were harvested, washed twice with PBS, re-suspended in 0.5 ml of ice-cold PBS, and fixed with 4 ml of ice-cold 70% ethanol for 48 h. Untreated cells cultured under the same conditions served as negative controls. Cells were then pelleted, washed twice with ice-cold PBS, re-suspended, and incubated at room temperature in 0.2 ml of staining buffer in the dark supplemented with 50 μg of RNAse and propidium iodide (PI; 50 μg/ml of final concentration). The distribution of cell cycle phases with different DNA contents is determined by flow cytometry (AccuriTM C6; Becton, Dickinson, and Company). Analysis of cell cycle distribution and percentage of cells in sub-G1, G1, S, and G2/M phases of the cell cycle were determined using the cell cycle platform of the FlowJo software with the Watson pragmatic model (Tree Star).

### Assessment of Apoptosis by Annexin V- PI Flow Cytometry

Chondrocytes were seeded at a density of 210,000 cells/cm^2^. Cells were harvested, washed twice with PBS, stained for 20 min with 0.2 ml of staining buffer containing annexin (Annexin V-FITC Apoptosis Staining/Detection Kit; Abcam, United States) in the dark. Cells were then analyzed for apoptosis by flow cytometry (Accuri C6; Becton Dickinson) at 488 nm excitation; a 530/30 nm band pass filter for fluorescein detection, and a long pass filter 670 nm. Annexin V^+^ cells were considered as apoptotic/necrotic; PI^+^ were counted as showing late apoptosis or necrosis, and Annexin V^+^ PI^−^ cells were counted as early apoptotic cells. Untreated cells cultured under the same conditions served as negative controls. Flow data were then analyzed by FlowJo software with the Watson pragmatic model (Tree Star, Ashland OR, United States).

### Immunofluorescence

The C-20/A4 chondrocytes were seeded with an approximate density of 263,000 cells/cm^2^ in sterile 12-well culture plates on the coverslips pre-coated with poly-l-lysine. After treatment with 200 or 300 µM FAC for 24 and 48 h, the cells were washed with PBS and fixed with paraformaldehyde (4%) at room temperature for 15 min. Post fixation cells were permeabilised for 10 min by treatment with 0.1% Triton X-100. Later, cells were blocked with 3% BSA for 1 h, rinsed with 1X PBS, and incubated at 4°C overnight with primary antibodies diluted at 1:500. The primary antibodies used were anti-Collagen II antibodies: (Cat No. ab185430) from LifeSpan Biosciences, Seattle, Washington. Cells were then washed with 1X PBS and incubated for an hour at 37°C with Alexafluor^®^680-labelled (Abcam) secondary antibodies, rinsed with 1X PBS. To stain the nuclei, mounting medium with ProLong gold antifade mountant with 4′,6′- diamidino-2-phenylindole (DAPI) (Invitrogen, Carlsbad, CA, United States) was used. The cells were visualised by using an Olympus BX51 fluorescence microscope (Olympus Corporation, Tokyo, Japan). The images were analysed for quantification of collagen II levels by using ImageJ software (Schneider et al., 2012).

### Statistical Analysis

Data analyses were conducted using GraphPad Prism 8 (GraphPad Software Inc., La Jolla, CA, United States); Two-way ANOVA with post-hoc Tukey’s test was used to compare between groups. *p* ≤ 0.05 was considered a significant difference; an asterisk (*) and dollar (^$^) denote statistical significance in cases where groups were compared with controls at 24 and 48 h time points respectively and a hash (^#^) symbol denotes significance when treatment groups were compared to other treatment groups from different time points. Three independent experiments meant that the experiments were run in a sequence to obtain reproducible results.

## Results

### FAC treatment reduces C-20/A4 cell viability

**FIGURE 1:**
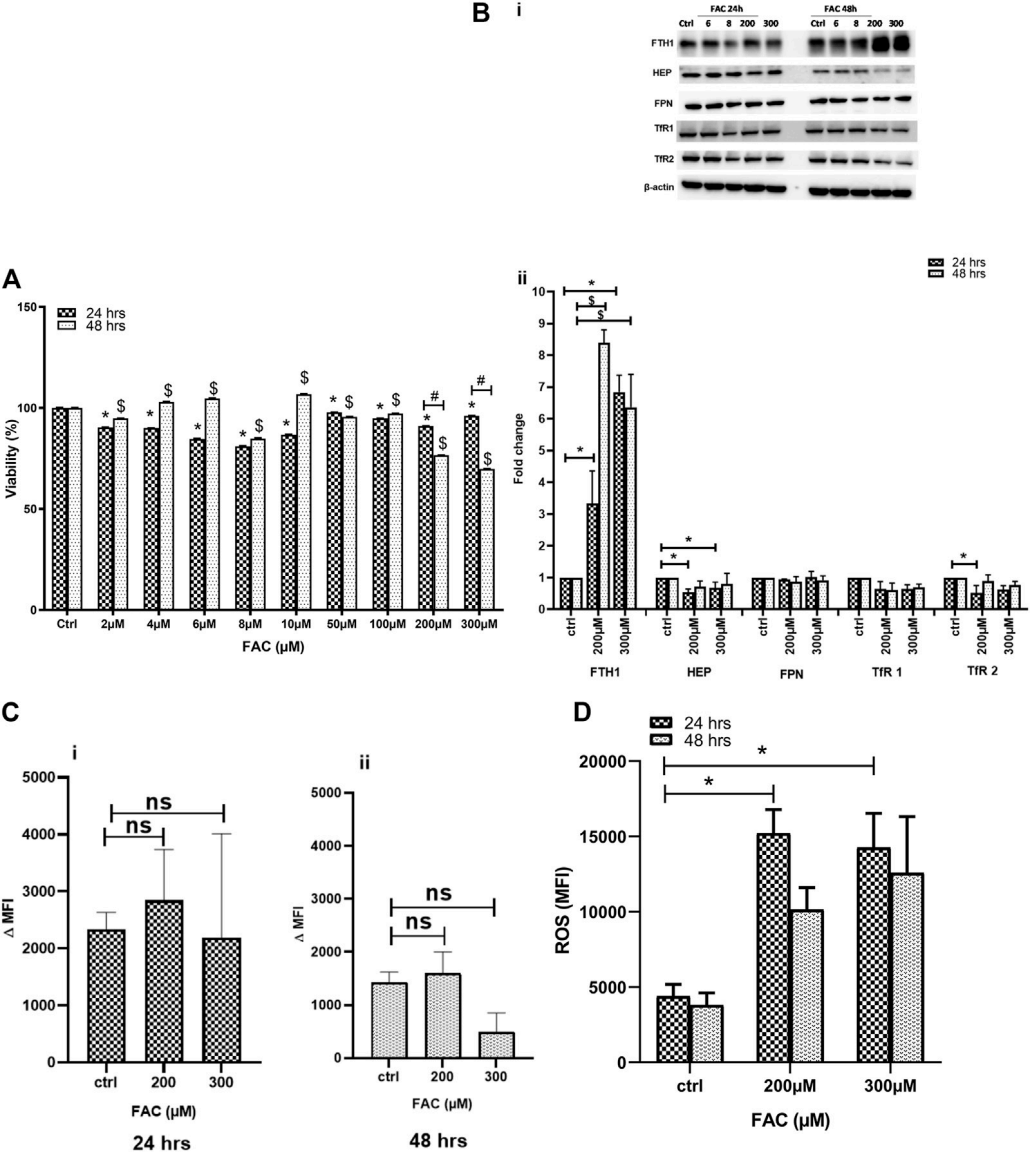
**(A)** FAC treatment reduces C-20A/4 cell viability. The viability of C-20A/4 chondrocytes was assessed using the MTT assay following treatment with 2, 4, 6, 8, 10, 50, 100, 200 or 300 µM of FAC for 24 or 48 h. Data are the mean ± SEM of three independent experiments. **(B)** Expression status of key IRGs in FAC-treated C-20A/4 cells. **(Bi)** Western blotting was used to examine the expression status of some key IRGs, including FTH1, HEP, FPN, TfR1 and TfR2 using lysates obtained from C-20A/4 cells treated with 200 or 300 µM FAC for 24 or 48 h. Untreated C-20A/4 cells cultured under similar conditions served as negative controls; β-actin was used as an internal loading control. **(Bii)** Fold change ±SD of protein band thickness based on three separate experiments. **(C)** FAC treatment and size of the labile iron pool (LIP) in C-20A/4 cells. Calcein staining flow cytometry-based analysis was used to determine the LIP content in C-20A/4 cells treated with 200 and 300 µM FAC for 24 and 48 h. Data shown is ΔMFI ±SD based on three independent experiments; *ns* means not significant. **(D)** Oxidative stress levels in FAC-treated C-20A/4 cells. To assess the level of oxidative stress under conditions of iron overload, ROS production was measure in C-20A/4 cells following treatment with 200 and 300 µM FAC for **(Ci)** 24 and **(Cii)** 48 h. Data shown are the mean ROS level ±SD based on three independent experiments. * (Treated vs*.* untreated control at 24 h), $ (Treated vs*.* untreated control at 48 h) and *#* (Treated at 24 h vs*.* treated at 48 h) denotes statistical significance at *p* <0.05; untreated cells cultured under the same conditions served as negative controls.

Previous work has suggested that excess iron causes chondrocyte death *in vivo* (van Vulpen et al., 2015a). In this preliminary set of experiments, we evaluated the impact of iron overload on chondrocyte viability *in vitro* using the FAC-treated human chondrocyte cell line C-20/A4. Cell viability was assessed in cultures treated with a wide range of FAC concentrations (6–300 µM) for 24 and 48 h. As shown in Figure 1A, C-20/A4 cells treated with increasing concentrations of FAC showed a significant reduction in cell viability, especially at 200–300 μM and 24 (25%) or 48 h (30%) post-treatment. Cell viability in cultures receiving low doses of FAC (2–100 µM) was also reduced after 24 h but showed an increase after 48 h post-treatment or was like that of controls.

### FAC Treatment Disrupts Cellular Iron Metabolism in C-20/A4 Cells

To further investigate the effect of FAC treatment on cellular iron metabolism, the expression status of key iron regulatory genes (IRGs) was investigated in C-20/A4 cells treated with increasing concentrations of FAC for 24 and 48 h. FAC-treated C-20/A4 cells exhibited a significant increase (six to eight folds) in the ferritin heavy chain (FTH1) expression, especially at high concentrations (200 and 300 µM) at both 24 and 48 h (Figure 1Bi, top panel and Figure 1Bii). Moreover, FAC-treated C-20/A4 cells showed a significant reduction in HEP synthesis at 24 h post-treatment with 200–300 µM (Figure 1Bi, second panel from top and Figure 1Bii). A comparable trend of reduction in the expression of TfR1 and TfR2 receptors was observed in cells treated with 200–300 µM for 24 and 48 h. No significant change was observed in FPN expression irrespective of FAC concentration or time point (Supplementary Figure S1).

To further investigate whether the profile of increased FTH1 and reduced HEP expression was reflective of iron sequestration or release, the labile iron pool (LIP), which represents the cellular ferrous iron content that drives metabolism and oxidative stress (Kruszewski, 2003), was measured in cells treated with 200–300 µM FAC for 24 and 48 h using the calcein-AM flow cytometry-based method. As shown in Figures 1Ci & 1Cii, treatment with FAC resulted in a slight and transient increase in LIP content in cells receiving FAC especially at 24 h as compared with control untreated cells.

### Iron Overload Precipitates Oxidative Stress and Associates With Chondrocyte Cell Death

**FIGURE 2:**
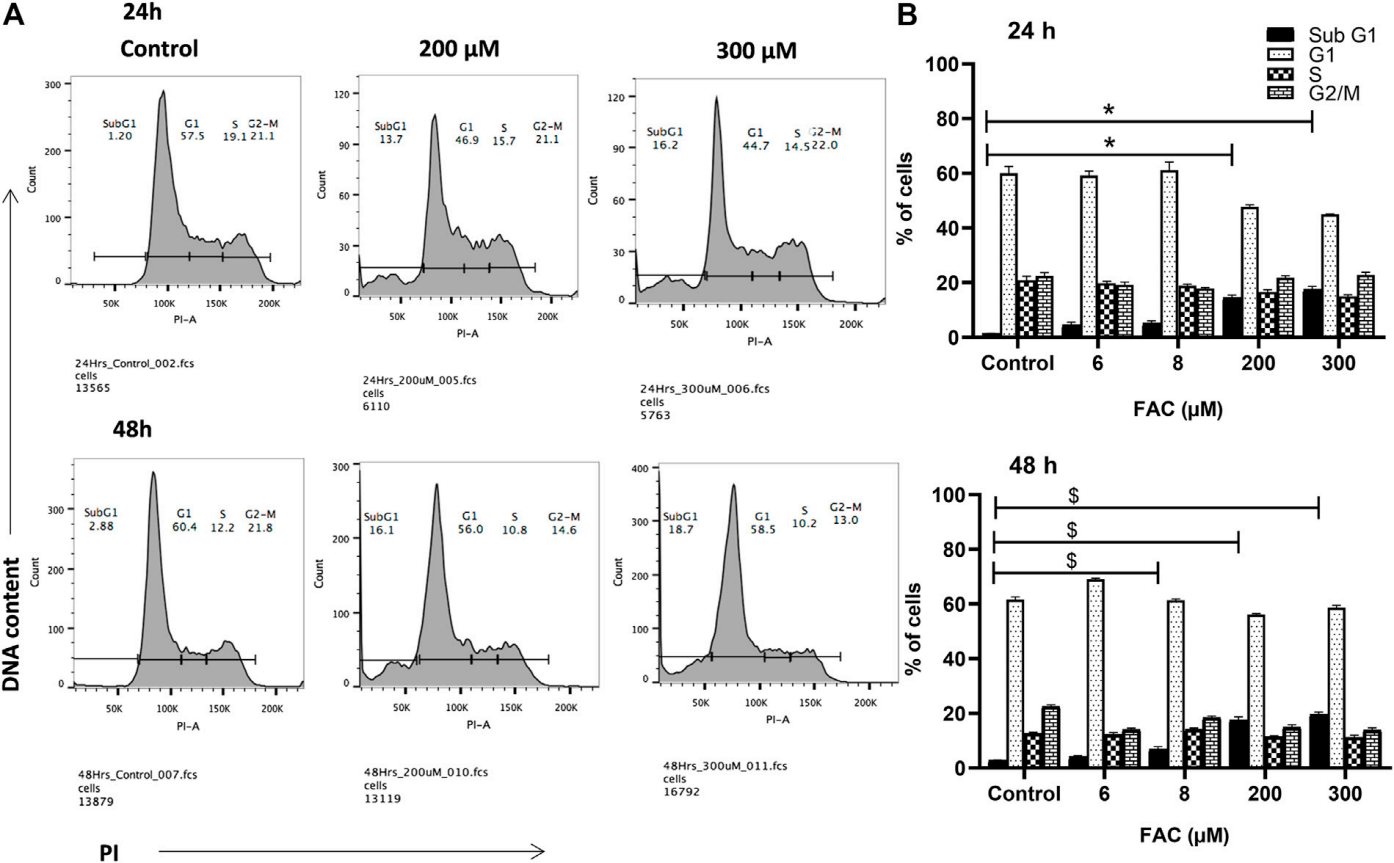
Iron overload and cell cycle progression analysis in chondrocytes. Cell cycle progression of C-20A/4 cells was examined following treatment with 200 and 300 µM of FAC and 24 or 48 h using the PI staining flow cytometry-based method. **(A)** Cell cycle profile in FAC-treated and control C-20A/4 cells; data shown are representative of three independent experiments. **(B)** Calculated distribution of cell-cycle-related subpopulations based on three independent experiments with ±SD; *,^$^ denotes statistical significance at *p*<0.05 as per the indicated group vs*.* its untreated control at 24 and 48 h respectively. Untreated cells cultured under the same conditions served as negative controls.

**FIGURE 3:**
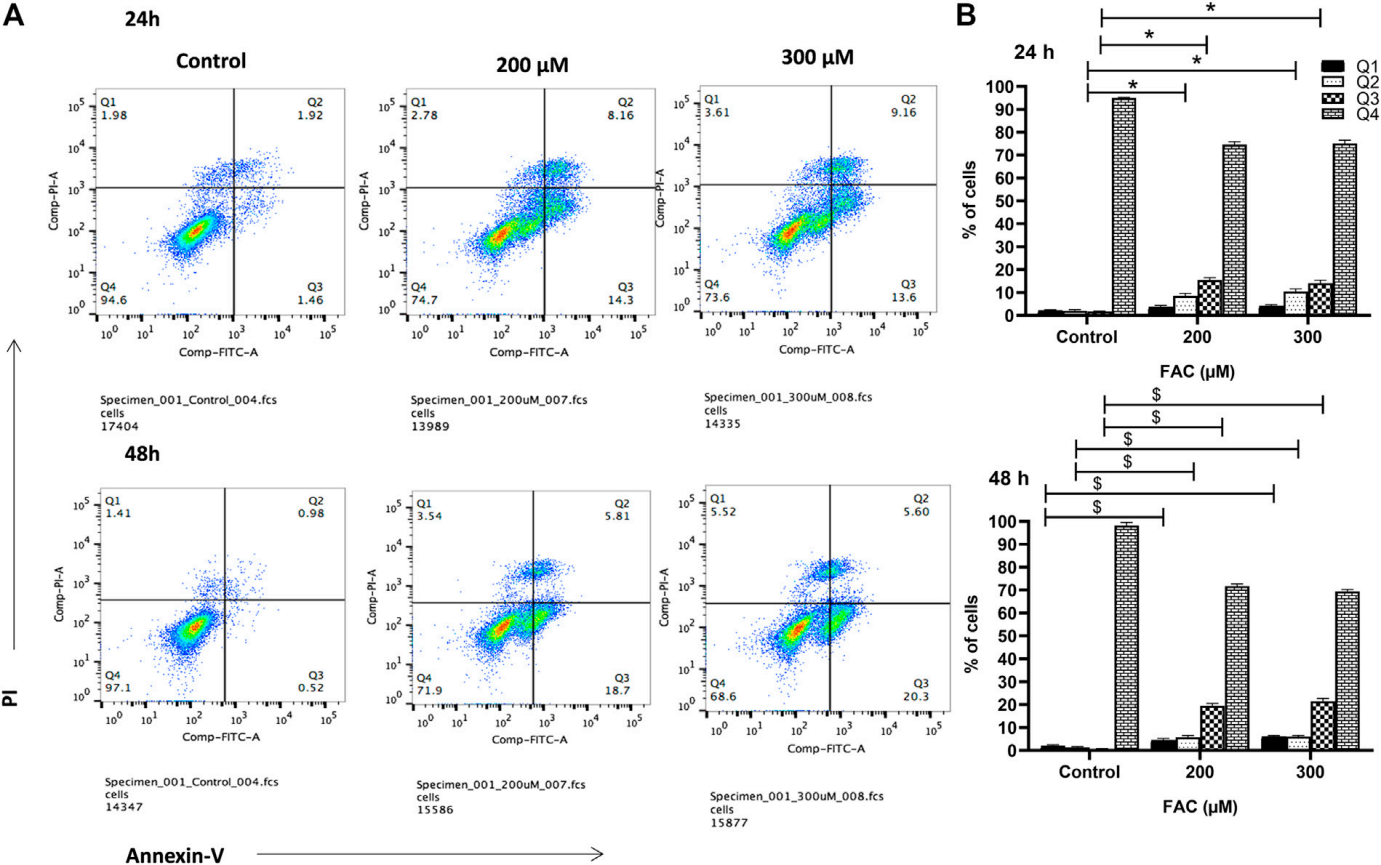
Iron overload induces apoptosis in chondrocytes. **(A)** Percentage pro-apoptotic and apoptotic C-20A/4 cells was examined following treatment with 200 or 300 µM of FAC and 24 or 48 h using the PI-annexin V staining-based flow cytometry method. **(B)** Mean ± SD percentage of pro-apoptotic and apoptotic C-20A/4 cells was calculated based on three independent experiments as in **(A)**. *,^$^ denotes statistical significance at *p*<0.05 as per the indicated group *vs.* its untreated control at 24 and 48 h respectively. Untreated cells cultured under the same conditions served as negative controls.

To investigate the consequences of iron overloading on chondrocyte survival, several parameters, including oxidative stress, cell cycle arrest and apoptosis were examined in FAC-treated C-20A/4 cells. As shown in Figure 1D, iron overload resulted in a significant (*p* < 0.05) and dose-dependent increase in ROS production in C-20A/4 cells. Consistent with cell viability data (Figure 1A), cell cycle analysis revealed a marked increase in sub-G1 C-20/A4 cells following treatment with 200 or 300 µM FAC for 24 or 48 h (Figure 2). Aside from the noticeable increase in sub-G1 cells, no significant shifts were observed in G1/S or G2/M phases of the cell cycle. To further validate the data observed in Figure 2, the percentage of pro-apoptotic and apoptotic chondrocytes was measured in FAC-treated C-20A/4 cells at 24 and 48 h post-treatment. As shown in Figure 3, an increase in the percentage of pro-apoptotic and apoptotic cells was evident at 24 and 48 h post-treatment with FAC.

### Iron Overload Decreases Collagen II Production in Chondrocytes

**FIGURE 4:**
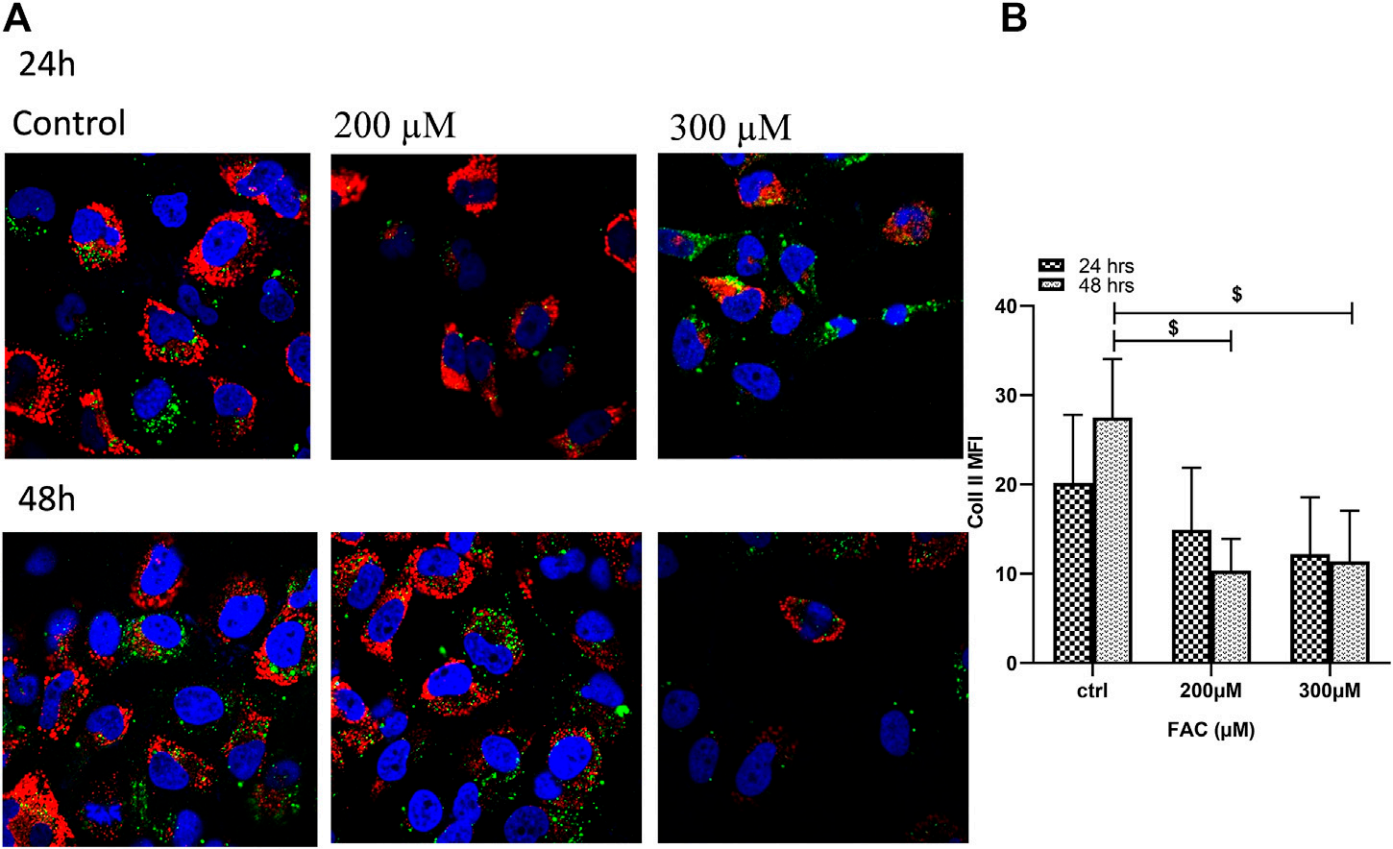
Iron overload decreases collagen II production in chondrocytes. **(A)** Expression of collagen II by chondrocytes treated with FAC (200 or 300 µM) and 24 or 48 h were assessed by immunofluorescence microscopy. Collagen II was labelled with Alexafluor^®^680 (Red), and nuclei were labelled with DAPI (Blue). Images were taken at ×100 magnification. **(B)** Expression levels of collagen II by FAC treated chondrocytes in comparison to the controls shown as represented in the graph. Data shown are representative of three independent experiments. $ denotes statistical significance at *p*<0.05 as per the indicated group *vs.* its untreated control at 48 h.

To evaluate the consequences of iron overload on the production of extracellular matrix proteins by chondrocytes, synthesis of collagen II was examined in FAC-treated C-20A/4 cells and compared with that in untreated controls. As shown in Figure 4, iron overload resulted in a significant (*p* < 0.05) and dose-dependent decrease in collagen II production by C-20A/4 cells.

## Discussion

The role of iron in the initiation and/or development of OA remains unclear. We found that chondrocytes with iron overload demonstrated increased cell death, a hallmark of OA pathogenesis. Moreover, we observed that chondrocytes treated with higher concentrations of iron, mediated disrupted iron homeostasis, and played a pivotal role in cell damage and eventually cell death. We also demonstrated that increased labile iron content led to the production of higher levels of oxidative stress, reduced collagen II expression, and disrupted cell cycle.

Clinical evidence has long suggested a potential link between local iron accumulation and increased susceptibility to OA-like arthropathy (Schumacher, 1982; van Vulpen et al., 2015b). Moreover, ferroptosis, a form of cell death that associates with iron accumulation, has been recently shown to associate with cartilage degeneration in OA (Yao et al., 2021). Adverse effects of systemic iron overload on joint health have been reported in several experimental studies (Simão et al., 2019; Burton et al., 2020). However, the question of whether local iron accumulation in the vicinity of joints is linked to systemic iron levels is still unanswered. Several studies have also suggested that iron chelation does not improve the symptoms of OA suggesting perhaps that iron accumulation leads to permanent tissue damage (Kra et al., 1965; Askari et al., 1983). Additionally, epidemiological studies have reported higher serum iron levels in elderly women compared to young counterparts (Wang et al., 2016), in post-menopausal women as compared to pre-menopausal women and in middle-aged and elderly men as compared to adolescents (Kim et al., 2012). However, clearly not all older people will develop OA. Therefore, it is possible that localized iron accumulation in cartilage and specifically in chondrocytes and disruption of chondrocyte iron metabolism may be a factor which, along with others, trigger joint damage leading to OA-like arthropathy. Data regarding iron homeostasis in healthy chondrocytes and dysregulation in OA are minimal.

Our data suggested that exposing chondrocytes to high iron levels results in increased FTH1 expression and reduced HEP and TfR1 & TfR2 expression. This is consistent with the results reported by a recent study that investigated the effects of systemic iron overload on OA resistant guinea pig strain 13 where high levels of the iron storage protein ferritin and low levels of the iron importing protein TfR1 were observed (Burton et al., 2020). Our results are also consistent with the observation that iron overload associated with upregulated FTH1 expression and decreased TfRs expression in mouse primary chondrocytes (Simão et al., 2019). Furthermore, it has been reported that dysregulation of iron homeostasis due to iron overload associates with cartilage degeneration possibly due to increased oxidative stress (Jing et al., 2020; Jing et al., 2021). Changes in the expression pattern of IRGs observed in chondrocytes in our study are reflective of the well documented compensatory response of mammalian cells mount under conditions of iron overload as a means of restoring iron homeostasis (Hentze et al., 2004; Anderson et al., 2012). In the present study, we found that when iron was in excess, chondrocytes had the capacity to store iron as holoferritin, but only up to a limit. It is worth remembering here that high FTH1 levels lead to TfRs and thereby reduce iron import into cells. That said, it is possible that once the normal iron storing capacity is exceeded, increased LIP content may precipitate oxidative stress and cell damage. This is consistent with the observation that iron overloaded chondrocytes had higher LIP levels and exhibited disrupted cellular iron homeostasis leading to higher levels of oxidative stress, cell cycle disruption, and eventually inducing apoptosis. This is in line with previous reports which have suggested that increased LIP content promotes the formation of ROS in mammalian cells (Comporti, 2002). Our findings are also consistent with a recent study which has shown that iron overload induces ferroptosis, which damages the joint tissues (Yao et al., 2021). Given the considerable cross-talk between ferroptosis and other forms of cell death including apoptosis (Hong et al., 2017; Neitemeier et al., 2017), it is possible that molecular events precipitated by ferroptosis may have contributed to the apoptotic events in iron-overloaded chondrocytes.

High levels of ROS have been identified to play a potential role in the pathophysiology of OA (Eisenstein, 2000; Suantawee et al., 2013). Furthermore, iron overload was reported to associate with high ROS production and deposition of (calcium phosphate, hydroxyapatite and/or urate) crystals in the OA joints, thereby exacerbating joint pathology (Naughton, 2001). Moreover, ROS plays a pivotal role in inducing chondrocyte apoptosis in OA (DelCarlo and Loeser, 2006), a hallmark of this disease. Recently, ferroptosis, a specific form of cell death caused by iron accumulation (Kuang et al., 2020), has been reported to precipitate cell death and cartilage degeneration in OA (Yao et al., 2021). We further investigated the detrimental effects of iron accumulation on the metabolic properties of chondrocytes. Degradation of collagen type II is a well-recognized feature in the development of OA (Poole et al., 2002). Our experiments revealed that excess iron lead to decreased production of collagen II in chondrocytes. This is consistent with the observation that a similar reduction in collagen type II was observed in iron overloaded animals associated with cartilage degeneration (Burton et al., 2020). Therefore, it is clear that iron accumulation leads to cartilage degeneration and the development of OA as the ultimate outcome. That said, the fact that this study has utilized a single immortalized chondrocyte cell line that was originally derived from ribcage cartilage is a limitation; hence, the observed effects of iron overload on chondrocyte health need to be validated by future *in vitro* and/or *in vivo* studies.

We conclude that iron overload is a likely detrimental factor for the progression of OA-like arthropathy. Consistent with previously published data (Yao et al., 2021), our data has clearly demonstrated that iron overload disrupts iron homeostasis, induces oxidative stress, promotes apoptosis and compromises the functional competence of chondrocytes. Taken together, these findings suggest that iron accumulation in chondrocyte may represent a potential therapeutic target in OA-like arthropathy.

## Data Availability

The raw data supporting the conclusion of this article will be made available by the authors, without undue reservation.
