# Supplementary figures and images for: Iron Overload Induces Oxidative Stress, Cell Cycle Arrest and Apoptosis in Chondrocytes
Source: Front Cell Dev Biol. 2022 Feb 18;10:821014. doi: 10.3389/fcell.2022.821014 (PMC8894434; doi:10.3389/fcell.2022.821014)

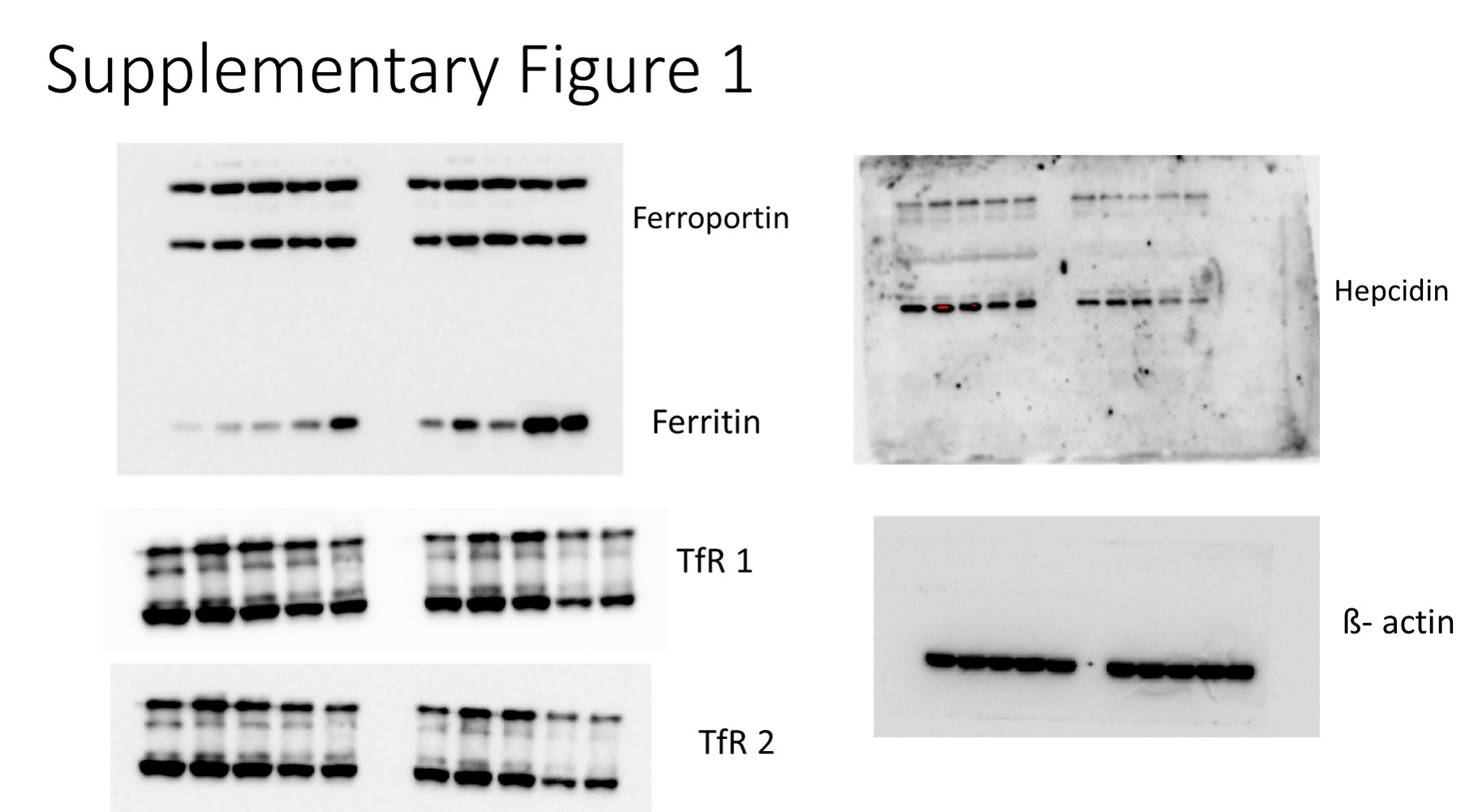

Supplement: Supplementary file 1 [file Image1.jpg]
